# Optimal Deployment Strategy for Reconfigurable Intelligent Surface under LoSD via Joint Active and Passive Beamforming

**DOI:** 10.3390/e25071073

**Published:** 2023-07-17

**Authors:** Ke Zhao, Zhiqun Song, Jun Xiong

**Affiliations:** 1 The 54th Research Institute of CETC, Shijiazhuang 050081, China; zhaoke_sx@163.com; 2 Science and Technology on Communication Networks Laboratory, Shijiazhuang 050081, China; 3 College of Electronic Science and Technology, National University of Defense Technology, Changsha 410073, China; xj8765@nudt.edu.cn

**Keywords:** RIS, LoSD, alternating optimization, deployment strategy

## Abstract

A reconfigurable intelligent surface (RIS) is a new and revolutionizing technology to achieve spectrum-efficient (SE) and energy-efficient (EE) wireless networks. In this paper, we study an optimal deployment strategy of RIS in a line-of-sight domain (LoSD) based on an actual deployment scenario, which jointly considers path loss, transmit power and the energy efficiency of the system. Furthermore, we aim to minimize the transmit power via jointly optimizing its transmit beamforming and the reflect phase shifts of RIS, subject to the quality-of-service (QoS) constraint, namely, the signal-to-noise ratio (SNR) constraint at the user. However, this optimization problem is non-convex with intricately coupled variables. To tackle this challenge, we first apply proper transformation on the QoS constraint and then propose an efficient alternating optimization (AO) algorithm. Simulation results demonstrate that compared to a conventional endpoint deployment strategy that simply deploys RIS at the transceiver ends, our proposed LoSD deployment strategy significantly reduces the transmit power by optimizing the available LoS links when a single RIS is relayed. The impact of the number of reflect elements on the system EE is also unveiled.

## 1. Introduction

While 5G is yet to be realized fully, researchers have already started looking for energy- and spectral-efficient solutions for 6G systems. In addition to EE and SE, the new paradigm is smart and reconfigurable wireless environments [1,2,3]. In traditional communication networks, an improvement in system performance focuses on the transceiver design, while the wireless channel which carries the signal propagation is usually regarded as an uncontrollable environmental factor. However, the generalized Snell’s law put forward in 2011 proved that the propagation direction of electromagnetic waves can be controlled by introducing phase discontinuity of spatial gradient at the interface [4]. In 2014, Professor Cui of Southeast University put forward the concept of “coding metamaterial” [5]. Through coding metamaterial, the modulation of the continuous domain is innovatively converted into the digital domain by discretizing continuous electromagnetic parameters using binary coding. Thus, coding metamaterial can empower smart wireless environments by overcoming the stochastic nature of the propagation channel, thereby improving QoS and connectivity, and it is also called RIS [2]. RIS is a planar array consisting of a large number of sub wavelength electromagnetic elements, which are reconfigurable and passive. The electromagnetic characteristics of RIS can be controlled dynamically by controlling the voltage of the elements, and finally, the active regulation of space electromagnetic waves is realized. Its flexible manipulation of electromagnetic waves satisfies people’s assumption of controlling wireless channels. As a two-dimensional representation of three-dimensional metamaterials, an RIS has the characteristics of software programmability, low cost, low complexity, low power consumption and easy deployment. The introduction of RIS makes the wireless communication environment change from passive adaptation to active control, thus building an intelligent wireless environment [6].

At present, the research direction of RIS-aided communication systems mainly focuses on channel estimation, reflection modulation and the deployment strategy [7]. The path loss in the system seriously affects the performance of the communication link because the reflect elements of RIS are usually passive. Meanwhile, the deployment location and routing of RIS also determine the upper limit of RIS-aided system performance, so the deployment strategy of RIS is the most important and basic problem. The authors of [8] maximized the received signal power by jointly optimizing the active and passive signals received by users, and it is concluded that the closer the relative position of an RIS and users is, the greater the received power is. Furthermore, it is proved that the performance of centralized deployment is better than that of distributed deployment. In the case of distributed deployment, the number of reflect elements needs to be optimized according to the different relative positions of the RIS [9]. The authors of [10] discussed the deployment position of RIS through the blocking probability, and concluded that the blocking probability is higher when an RIS is deployed in a place that is particularly far or very close to the base station. RIS can also be deployed on unmanned aerial vehicles (UAV) for anti-jamming communication, and the trajectory of RIS and passive beamforming in airspace are jointly optimized using an alternating optimization algorithm [11]. In [12], the authors reconstructed the composite channel gain by taking the rotations at the RIS plane, transmit antenna and receive antenna into account, and investigating the RIS deployment strategy. To analyze the coverage of a downlink RIS-assisted network with one base station (BS) and one piece of user equipment (UE), the authors in [13] proposed a coverage maximization algorithm (CMA) to maximize cell coverage by optimizing RIS orientation and horizontal distance.

Single RIS-assisted communication systems are discussed in the above research. However, users always need to be in their reflection half-space due to the limited coverage of an RIS with single RIS-assisted systems, and one RIS may not be able to provide sufficient channel gain due to its size limitation. Cascade reflection links with two RIS are studied in [14], and it is proposed that with the increase in the number of reflect elements, a dual reflection link can bring the channel gain to fourth power, while the channel gain brought by two RIS can make up for the extra path loss when the number of RIS elements is large enough and the distribution is reasonable. A multiple RIS-assisted communication link was discussed in [15], which is similar to the conclusion in [14]. The key to multi-RIS cooperative work is to balance the cooperative passive beamforming, so as to maximize the gain and minimize the path loss. Two of them play different leading roles when the number of reflect elements is different. In order to enhance the coverage performance of wireless networks, the authors in [16] divided the coverage area of the network into multiple non-overlapping cells and formulated a joint BS and RIS deployment problem based on graph theory.

However, most of the above discussions about the deployment strategy of RIS are based on the ideal situation. The scenario assumption is relatively simple, and the factors such as space constraints and the availability of LoS channels in actual deployment are not fully considered. This paper firstly proposes the concept of an LoSD deployment strategy based on an RIS-aided MISO communication system, and obtains the optimal deployment coordinates of RIS by searching the potential deployment positions. Based on the optimal deployment position in LoSD, active and passive beamforming are jointly optimized using an alternating optimization algorithm, so that the transmission power is minimized under the condition of fixed SNR at the user. The total power consumption and energy efficiency of the system are also discussed in this paper.

## 2. System Model and Problem Formulation

### 2.1. System Model

**Figure 1:**
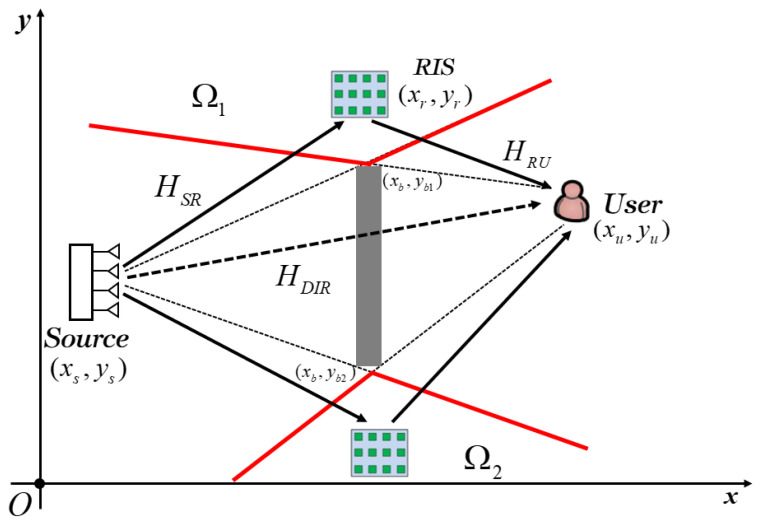
Single RIS-aided communication in an LoSD (divided into above bound and below bound).

We consider a single RIS-aided downlink communications system, where the RIS is deployed in an LoSD to assist communication links from a multi-antenna source to one single-antenna user, as shown in Figure 1. The transmit antennas are placed in a uniform linear array (ULA) on a wall with the distance *S_t_*, and the antenna array is parallel to the ground. The RIS is installed on a vertical wall in an LoSD, and the center of the RIS is at the same height as the source and the user. It consists of reflect elements placed in a uniform rectangular array (URA) with *N_a_* rows and *N_b_* columns (the total number of RIS reflect elements is *N*, where *N* = *N_a_N_b_*). Each element size is λ/2 × λ/2, where λ denotes the wavelength of operation. We assume that the reflect elements are in an ideal state, which means the amplitude and phase of each element respond independently. The coordinates of the midpoint at the source antenna arrays, the RIS elements and the user end are (*x_s_*, *y_s_*), (*x_r_*, *y_r_*) and (*x_u_*, *y_u_*), respectively. The direct link from the source to the user is blocked by the wall with an area of (*x_b_*, *y_b_*_1_ − *y_b_*_2_)(*y_b_*_1_ > *y_b_*_2_). In a single RIS-aided communication system, the potential LoSD deployment area of RIS is the intersection of LoSDs of source-RIS and RIS-user links, which is divided into above bound Ω_1_ and below bound Ω_2_, respectively. The optimal deployment location is the boundary of LoSDs, which are represented by the red lines in Figure 1.

The signal vector at the user receive antenna is given by

(1) y=Hx+n

where ***H*** is the transmission channel matrix, which includes the direct link from the source to the user and the RIS-aided reflect link. ***x*** = ***w****s* denotes the transmit signal vector at the source, where $w\in {\mathbb{C}}^{M\times 1}$ is the corresponding beamforming vector and ${\mathbb{C}}^{a\times b}$ denotes the space of a × b complex-valued matrices. It is assumed that *s* is a random variable with zero mean and unit variance, while *n* denotes the additive white Gaussian noise (AWGN) at the user with zero mean and $\sigma^{2}$ variance. We assume that the total average transmit power has a maximum value of *P_t_*, i.e., ${\left\|w\right\|}^{2}\leq P_{t}$, $\left\|\;\cdot \;\right\|$ denotes its Euclidean norm.

### 2.2. Channel Model

The channel matrix ***H*** in an RIS-aided communication system can be expressed as

(2) $$H=H_{DIR}+H_{INDIR}$$

where ***H****_DIR_* denotes the direct link between the source and the user, which can be divided into a blocking and non-blocking situation according to the path loss, and ***H****_INDIR_* is the virtual LoS link based on the RIS reflect link. Adopting the Rician fading channel model, the direct link channel matrix ***H****_DIR_* is given by

(3) $$H_{DIR}=\frac{\sqrt{\beta_{DIR}{}^{-1}}}{\sqrt{K_{SU}+1}}(\sqrt{K_{SU}}H_{DIR,LoS}+H_{DIR,NLoS})$$

where $H_{DIR,LoS}(m)=e^{-j2\pi d_{m}/\lambda }$ denotes the LoS component of the direct link, and *d_m_* is the distance between the *m*-th transmit antenna and the user. $H_{DIR,NLoS}$ denotes the NLoS component of the direct link which is independent and identically distributed (i.i.d.) with zero mean and unit variance. The free space path loss of the direct link is $\beta_{DIR}={\left(4\pi /\lambda \right)}^{2}d_{0}^{\alpha_{DIR}}$, where $d_{0}=\sqrt{{\left(x_{u}-x_{s}\right)}^{2}+{\left(y_{u}-y_{s}\right)}^{2}}$ denotes the distance between the source and the user, and $\alpha_{DIR}$ is the path loss exponent of the direct link, whose value is influenced by the obstacle present [17]. $K_{SU}$ is the Rician factor of the direct link, and its value is chosen to form the interval [0,+∞). The channel model becomes an LoS channel when the Rician factor approaches infinity, and becomes a Rayleigh fading channel when the Rician factor is 0. If the channel gain of the direct link is higher than that of the indirect link, the source should transmit the signal directly to the user; otherwise, it should transmit through the reflective link.

The reflect link channel matrix $H_{INDIR}$ can be written as

(4) $$H_{INDIR}=H_{SR}\Theta H_{RU}$$

where $H_{SR}\in {\mathbb{C}}^{N\times M}$ represents the channel between the source and the RIS, while $H_{RU}\in {\mathbb{C}}^{1\times N}$ represents the channel between the RIS and the user. The phase shift matrix of the reflect elements can be expressed as $\Theta =diag(\theta )\in {\mathbb{C}}^{N\times N}$, where $\theta ={\left[\theta_{1},\theta_{2},\ldots ,\theta_{N}\right]}^{T}\in {\mathbb{C}}^{N\times 1}$. Assuming that the reflection coefficient of the *n*-th reflect element on the RIS is $\theta_{n}=A_{n}e^{j\varphi_{n}}$, *n* = 1, 2, …, *N*, where $A_{n}\in [0,1]$ and $\varphi_{n}\in [0,2\pi )$ represent the reflect amplitude and phase shift of the *n*-th reflect element, respectively. In order to optimize the reflection performance, we assume that the amplitude and phase responses of reflect elements are independent of each other, and the reflect amplitude is fixed as 1.

(5) $$A_{n}=1,n=1,2,\ldots ,N$$

Adopting the Rician fading channel model, the source-RIS link channel matrix $H_{SR}$ is given by

(6) $$H_{SR}=\frac{\sqrt{\beta_{INDIR}{}^{-1}}}{\sqrt{K_{SR}+1}}(\sqrt{K_{SR}}H_{SR,LoS}+H_{SR,NLoS})$$

where $H_{SR,LoS}(n,m)=e^{-j2\pi d_{n,m}/\lambda }$ denotes the LoS component of the source-RIS link, and *d_n,m_* is the distance between the *m*-th transmit antenna and the *n*-th reflect element on the RIS. $H_{SR,NLoS}$ denotes the NLoS component of the source-RIS link which is independent and identically distributed (i.i.d.) with zero mean and unit variance. $K_{SR}$ is the Rician factor of the source-RIS link, and its value is chosen to form the interval [0,+∞).

Similarly, the RIS-user link channel matrix $H_{RU}$ is given by

(7) $$H_{RU}=\frac{1}{\sqrt{K_{RU}+1}}(\sqrt{K_{RU}}H_{RU,LoS}+H_{RU,NLoS})$$

where $H_{RU,LoS}(n)=e^{-j2\pi d_{n}/\lambda }$ denotes the LoS component of the RIS-user link, and *d_n_* is the distance between the *n*-th reflect element on the RIS and the user. $H_{SR,NLoS}$ denotes the NLoS component of the RIS-user link which is independent and identically distributed (i.i.d.) with zero mean and unit variance. $K_{RU}$ is the Rician factor of the source-RIS link, and its value is chosen to form the interval [0,+∞).

The free space path loss factor $\beta_{INDIR}{}^{-1}$ of the reflect link can be expressed as

(8) $$\beta_{\mathrm{INDIR}}^{-1}=\frac{\lambda^{4}}{256\pi^{2}}\frac{{\left(\cos\varphi_{1}+\cos\varphi_{2}\right)}^{2}}{d_{1}^{2}d_{2}^{2}}$$

where *d*_1_ and *d*_2_ represent the distance between the source and the RIS, and between the RIS and the user, respectively. *φ*_1_ is the angle between the incident wave direction from the transmit array midpoint at source to the RIS center and the vector normal to the RIS, and *φ*_2_ is the angle between the vector normal to the RIS and the reflect wave direction from the RIS center to the user [18].

### 2.3. Problem Formulation

There are some effective ways to obtain channel state information (CSI) [19,20,21], so we assume that the CSI of all involved channels is known perfectly. From Equations (1), (2) and (4), the received signal at the user is given by

(9) $$y=(H_{DIR}^{H}+H_{RU}^{H}\Theta H_{SR})ws+n$$

Accordingly, the SNR of the user is given by

(10) $$SNR=\frac{{\left|(H_{DIR}^{H}+H_{RU}^{H}\Theta H_{SR})w\right|}^{2}}{\sigma^{2}}$$

In this paper, we aimed to minimize the total transmit power at the source by jointly optimizing the active and the passive beamforming, subject to the SNR constraints of the user. It can be seen from Equation (10) that the transmit power is related to the channel matrix ***H*** while the user’s SNR is fixed, and ***H*** depends on the deployment position (path loss) of the RIS, the beamforming vector at the source and the phase shift matrix of the reflect elements. Accordingly, the optimization problem can be mathematically stated as

(11) $$(P1):\;\min_{(x_{r},y_{r}),w,\theta }\;{\left\|w\right\|}^{2}$$

(12) $$s.t.\;\frac{{\left|(H_{DIR}^{H}+H_{RU}^{H}\Theta H_{SR})w\right|}^{2}}{\sigma^{2}}\geq \gamma ,$$

(13) $$0\leq \theta_{n}\leq 2\pi ,\;n=1,2,\ldots ,N.$$

(14) $$(x_{r},y_{r})\in \Omega_{1}\cup \Omega_{2}$$

where γ is the minimum SNR requirement of the user. Although the objective function of (P1) and constraints in (13) and (14) are convex, the whole optimization problem is still non-convex due to the coupling between the transmit beamforming and phase shifts in (12). In this paper, we firstly searched for the optimal deployment position of the RIS in the LoSD, and then jointly optimized the transmit beamforming vector and RIS phase shift matrix based on the AO algorithm.

## 3. Optimal Deployment Strategy in the LoSD

The free space path loss of the direct link and reflect link are represented by $\beta_{DIR}$ and $\beta_{INDIR}$, respectively, and from Equation (8), it is shown that their value is directly proportional to the distance between the two nodes. The path loss of the direct link is fixed because the distance between the source and the user is fixed. It can be seen from Figure 1 that the optimal deployment position of RIS in potential LoSDs can be divided into four situations, and the relationship of the RIS coordinates (*x_r_*, *y_r_*) can be obtained from the geometric relationship.

(15) $$\left\{\begin{array}{l} y_{r}=(\frac{y_{u}-y_{b_{1}}}{x_{u}-x_{b}})(x_{r}-x_{b})+y_{b_{1}}\ldots \ldots \ldots (y_{r}>y_{b_{1}},x_{r}\leq x_{b})\\ y_{r}=(\frac{y_{s}-y_{b_{1}}}{x_{s}-x_{b}})(x_{r}-x_{b})+y_{b_{1}}\ldots \ldots \ldots (y_{r}>y_{b1},x_{r}>x_{b})\\ y_{r}=(\frac{y_{u}-y_{b_{2}}}{x_{u}-x_{b}})(x_{r}-x_{b})+y_{b2}\ldots \ldots \ldots (y_{r}<y_{b_{2}},x_{r}\leq x_{b})\\ y_{r}=(\frac{y_{s}-y_{b_{2}}}{x_{s}-x_{b}})(x_{r}-x_{b})+y_{b_{2}}\ldots \ldots \ldots (y_{r}<y_{b_{2}},x_{r}>x_{b}) \end{array}\right.$$

From the free space path loss of the cascaded link in Equation (8) and the path loss model in [22], it can be known that the virtual LoS link based on RIS-aided communication system has produced a path fading effect, which means the total path loss is proportional to the square of the distance product of two reflect links. In order to minimize the transmit power at the source with a fixed SNR constraint at the user, the distance product of the source-RIS link and RIS-user link is at a minimum with respect to the optimal location of the RIS in the LoSD. Thus, we have min *L*, where $L=d_{1}\times d_{2}$, $d_{1}=\sqrt{{\left(x_{r}-x_{s}\right)}^{2}+{\left(y_{r}-y_{s}\right)}^{2}}$ is the distance between the RIS and the source, and $d_{2}=\sqrt{{\left(x_{r}-x_{u}\right)}^{2}+{\left(y_{r}-y_{u}\right)}^{2}}$ is the distance between the RIS and the user. In order to obtain the minimum *L*, we searched for the optimal deployment position by moving the RIS in LoSDs, and then we obtained the minimum free space path loss and the maximum channel gain of the total cascaded link.

Assuming the location of the source, the user and the obstacle are (10,10) m, (110,40) m and (80,80:20) m, respectively. The distance product of the reflect links is divided into two cases: above LoSD and below LoSD, and we compared the LoSD deployment strategy with the endpoint deployment strategy in [7,8,9] at the same time.

**Figure 2:**
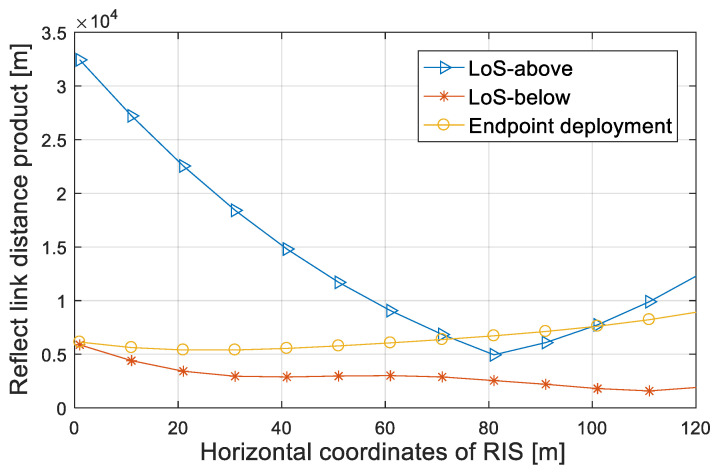
Product distance of reflection links versus the horizontal coordinates of RIS.

From the relationship between the reflect link distance product and the horizontal coordinates of the RIS in Figure 2, it can be seen that the endpoint deployment strategy has a local minimum when the abscissa is 25 m. Above the LoSD, the product of the reflect link has a local minimum when the abscissa is 80 m, and local minimum appears when the abscissas are 37 m and 110 m below the LoSD, while the latter is global minimum. The reflect link product is related to the source and user ends and the deployment position of the RIS. When the distance is large (above the LoSD), the optimal deployment position of RIS is near the upper limit of the obstacle. When the distance is small (below the LoSD), the optimal deployment position of RIS is not simply close to the transceiver ends or the midpoint, but related to the specific positions of the transceiver ends and the blocking area. Through the simulation results, the optimal deployment location of the RIS is (110,24) m. Based on the best location of the RIS, problem (P1) is simplified to

(16) $$(P2):\;\min_{w,\theta }\;{\left\|w\right\|}^{2}$$

(17) $$s.t.\;{\left|(H_{DIR}^{H}+H_{RU}^{H}\Theta H_{SR})w\right|}^{2}\geq \gamma \sigma^{2},$$

(18) $$0\leq \theta_{n}\leq 2\pi ,\;n=1,2,\ldots ,N.$$

Although the position of the RIS has been simplified, problem (P2) is still a non-convex optimization problem, since constraint (17) is not jointly concave with respect to ***θ*** and ***w***. In the next section, we use an alternating optimization technique to solve (P2) by jointly optimizing passive beamforming and active beamforming.

Without loss of generality, we considered the system model under the condition of a random distribution of the source, the user and the obstacle. As we can see from Figure 1, the potential deployment area of the RIS in the LoSD depends on the relative spatial position between the transceiver and the obstacle.

**Figure 3:**
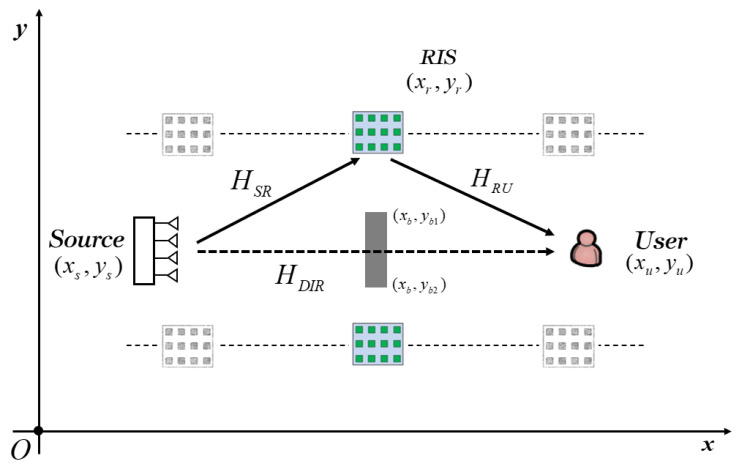
Single RIS-aided communication system model with a symmetrical source and user when the obstacle coverage is relatively small.

As shown in Figure 3, when the source and the user are symmetrical and the length of the obstacle coverage is less than a critical value *ξ*, i.e., *y_b_*_1_ − *y_b_*_2_ ≤ *ξ*, where *ξ* is usually less than the beam width in the farfield. Thus, the potential deployment area of RIS is parallel to the source and the user, and the optimal deployment scheme is similar to the endpoint deployment.

**Figure 4:**
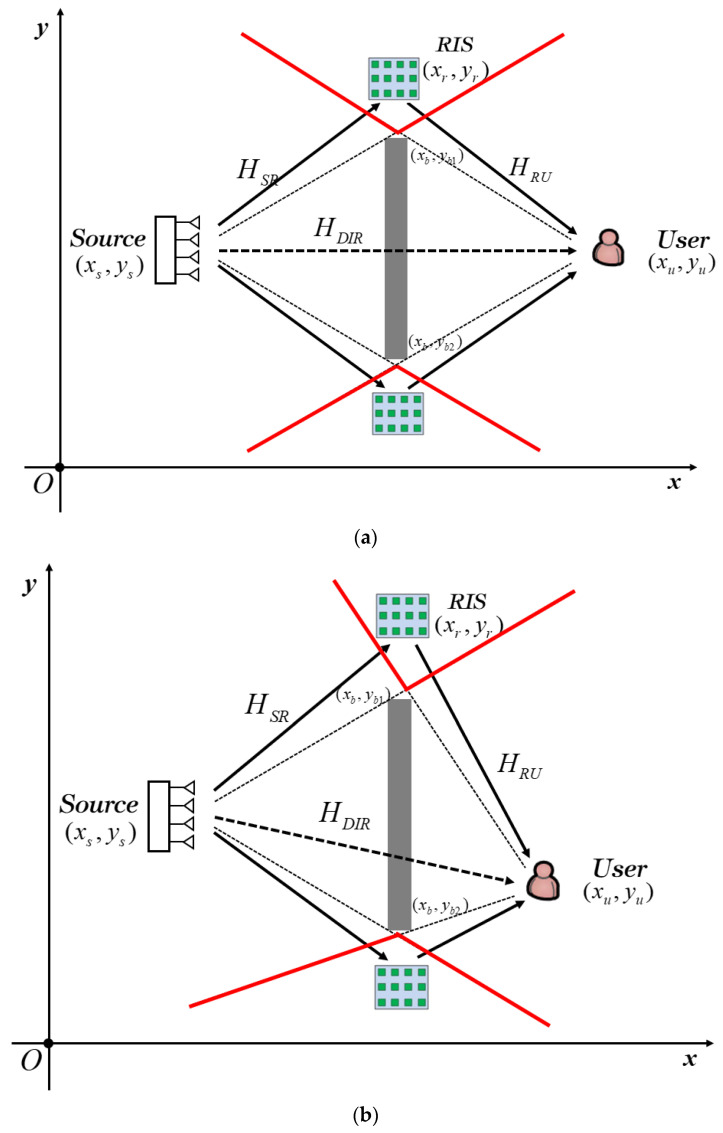
Single RIS-aided communication system model with a relatively large obstacle. (**a**) The source and the user are symmetrical. (**b**) The source and the user are asymmetrical.

As we can see from Figure 4, the potential deployment area of RIS is the intersection of the LoSDs of the source and the user when *y_b_*_1_ − *y_b_*_2_ ≫ *ξ*. The positions of the two nodes can be divided into symmetric and asymmetric situations, as shown in Figure 4a and Figure 4b, respectively. The optimal deployment position of RIS depends on the relative distance and angle between the source/user and the obstacle, which can be described by the geometric relationship in Equation (15).

## 4. Jointly Optimizing the Passive Beamforming and Active Beamforming

Let $w=\sqrt{P}\bar{w}$, which means the transmit beamforming vector includes transmit power *P* and transmit beamforming direction $\bar{w}$. With fixed $\bar{w}$, problem (P2) is reduced to a joint transmit power *P* and phase shift ***θ*** optimization problem (P3).

(19) $$(P3):\;\min_{P,\theta }\;P$$

(20) $$s.t.\;P\;{\left|(H_{DIR}^{H}+H_{RU}^{H}\Theta H_{SR})\bar{w}\right|}^{2}\geq \gamma \sigma^{2},$$

(21) $$0\leq \theta_{n}\leq 2\pi ,\;n=1,2,\ldots ,N.$$

From Equation (20), it is not difficult to verify that the transmit power satisfies $P\geq \frac{\gamma \sigma^{2}}{\;{\left|(H_{DIR}^{H}+H_{RU}^{H}\Theta H_{SR})\bar{w}\right|}^{2}}$. As such, our goal to minimize the transmit power is equivalent to maximizing the channel power gain; thus, problem (P3) is reduced to problem (P4).

(22) $$(P4):\;\max_{\theta }\;{\left|(H_{DIR}^{H}+H_{RU}^{H}\Theta H_{SR})\bar{w}\right|}^{2}$$

(23) $$s.t.\;0\leq \theta_{n}\leq 2\pi ,\;n=1,2,\ldots ,N.$$

Although the optimization problem (P4) is still non-convex, it can be solved with a closed-form solution by exploiting the special structure of its objective function.

(24) $$\begin{array}{l} \left|(H_{DIR}^{H}+H_{RU}^{H}\Theta H_{SR})\bar{w}\right|=\left|H_{DIR}^{H}\bar{w}+H_{RU}^{H}\Theta H_{SR}\bar{w}\right|\\ \;\overset{a}{\leq }\left|H_{DIR}^{H}\bar{w}\right|+\left|H_{RU}^{H}\Theta H_{SR}\bar{w}\right| \end{array}$$

where (a) is due to the triangle inequality and the equality holds if and only if $\arg(H_{RU}^{H}\Theta H_{SR}\bar{w})=\arg(H_{DIR}^{H}\bar{w})\overset{△}{=}\phi_{0}$. Let $H_{RU}^{H}\Theta H_{SR}\bar{w}=v^{H}a$, where $v={\left[e^{j\theta_{1}},\ldots ,e^{j\theta_{N}}\right]}^{H}$ and $a=\mathrm{diag}(H_{RU}^{H})H_{SR}^{H}\bar{w}$. With (24), the optimization problem (P4) is equivalent to problem (P5).

(25) $$(P5):\;\max_{v}\;{\left|v^{H}a\right|}^{2}$$

(26) $$s.t.\;\left|v_{n}\right|=1,\;n=1,2,\ldots ,N.$$

(27) $$\arg(v^{H}a)=\phi_{0}$$

The optimal solution for (P5) is given by

(28) $$v^{*}=e^{j(\phi_{0}-\arg(a))}=e^{j(\phi_{0}-\arg(\mathrm{diag}(H_{RU}^{H})H_{SR}^{H}\bar{w}))}$$

With the fixed transmit beamforming direction $\bar{w}$, the optimal phase shift of the *n*-th reflect element on the RIS is given by

(29) $$\begin{array}{l} \theta_{n}^{*}=\phi_{0}-\arg h_{RU,n}^{H}h_{SR,n}^{H}\bar{w}\\ \;=\phi_{0}-\arg(h_{RU,n}^{H})-\arg(h_{SR,n}^{H}\bar{w}) \end{array}$$

where $h_{RU,n}^{H}$ is the *n*-th element of $H_{RU}^{H}$ and $h_{SR,n}^{H}$ is the *n*-th row vector of $H_{RU}^{H}$.

The optimal phase shift of each reflect element on the RIS is shown in Equation (29), which suggests that the *n*-th phase shift should be tuned so that the communication link can achieve a coherent signal, combining at the user. Therefore, the SNR at the user can be improved and the transmit power can be reduced. As a result, the optimal transmit power $P^{*}$ is given by

(30) $$P^{*}=\frac{\gamma \sigma^{2}}{{\left\|(H_{DIR}^{H}+H_{RU}^{H}\Theta H_{SR})\bar{w}\right\|}^{2}}$$

Next, we optimized the transmit beamforming direction $\bar{w}$ with the fixed phase shift matrix $\Theta^{*}$ in (29). It is known that the maximum-ratio transmission (MRT) is the optimal transmit beamforming at the source [23]; thus, we have

(31) $$\bar{w*}=\frac{{\left(H_{RU}^{}\Theta H_{SR}^{H}+H_{DIR}^{H}\right)}^{H}}{\left\|H_{RU}^{}\Theta H_{SR}^{H}+H_{DIR}^{H}\right\|}$$

and the transmit beamforming vector $w^{*}$ is $\sqrt{p^{*}}\frac{{\left(H_{RU}^{}\Theta H_{SR}^{H}+H_{DIR}^{H}\right)}^{H}}{\left\|H_{RU}^{}\Theta H_{SR}^{H}+H_{DIR}^{H}\right\|}$.

### 4.1. System Total Power Consumption

With the fixed SNR γ, the transmit power *P* in direct link without an RIS is given by

(32) $$P=\frac{\gamma \sigma^{2}}{{\left\|H_{DIR}^{H}\right\|}^{2}}$$

Similarly, with the fixed SNR γ, the transmit power *P_RIS_* in an RIS-aided cascaded link is given by

(33) $$P_{RIS}=\frac{\gamma \sigma^{2}}{{\left\|(H_{DIR}^{H}+H_{RU}^{H}\Theta H_{SR})\bar{w^{*}}\right\|}^{2}}$$

Besides transmit power, the system’s total power consumption also includes hardware power consumption at the source and the user, and it also includes the control power consumption of the reflect elements in the RIS-aided communication link [24]. In the direct link without RIS, the system total power consumption can be represented as

(34) $$\;P_{\mathrm{total}}^{}=\frac{p}{\eta }+P_{s}+P_{u}$$

where η∈(0,1) denotes the transmit efficiency at the source, and *P_s_* and *P_u_* are the hardware power consumption at the source and the user, respectively. Assuming that the control power consumption of each reflect element is *P_e_*, the total power consumption is therefore given by

(35) $$\;P_{\mathrm{total}}^{\mathrm{RIS}}(N)=\frac{p_{\mathrm{RIS}}(N)}{\eta }+P_{s}+P_{u}+NP_{e}$$

### 4.2. System Energy Efficiency

We considered the system energy efficiency based on an RIS-aided communication link when the direct link is blocked. *EE* can be expressed as $B\cdot R/P_{total}^{RIS}$, where *B* is the operation bandwidth and $R=\log_{2}(1+\gamma )$ is the achievable rate at the user, i.e., channel capacity. The total system power consumption in an RIS-aided communication link is given by

(36) $$\;P_{total}^{RIS}(N)=\frac{\gamma \sigma^{2}}{\eta \cdot {\left\|H_{RU}^{H}\Theta H_{SR}\right\|}^{2}}+P_{s}+P_{u}+NP_{e}$$

The *EE* is given by

(37) $$\;EE=B\cdot (\log_{2}(1+\gamma ))/(\frac{\gamma \sigma^{2}}{\eta \cdot {\left\|H_{RU}^{H}\Theta H_{SR}\right\|}^{2}}+P_{s}+P_{u}+NP_{e})$$

Assuming that the RIS is in the optimal deployment position in the LoSD, and the phase shift is optimal ($\theta_{n}^{*}=\phi_{0}-\arg(h_{RU,n}^{H})-\arg(h_{SR,n}^{H}\bar{w})$), Equation (37) is equivalent to the following:

(38) $$\;EE=B\cdot (\log_{2}(1+\gamma ))/(\frac{\gamma \sigma^{2}}{\eta \cdot (\sum_{n=1}^{N}\left\|h_{RU,n}^{H}h_{SR,n}^{H}\right\|{)}^{2}}+P_{s}+P_{u}+NP_{e})$$

It can be seen from Equation (38) that the energy efficiency of the system changes with the number of RIS reflect elements under the condition that the SNR is fixed.

## 5. Numerical Results

**Figure 5:**
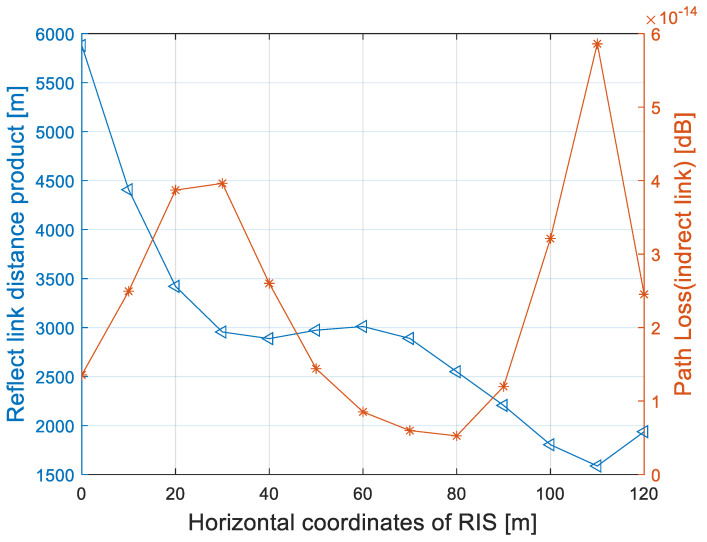
Distance product and path loss of the reflect link versus the horizontal coordinates.

In this section, numerical examples are provided to validate the effectiveness of the proposed LoSD deployment strategy. The number of reflect elements N and the transmit antennas M were set at 20 × 20 = 400 and 8, respectively, while the distance between the two antennas (elements) was λ/2. The noise power at the user was −120 dBm, and $\alpha_{DIR}$ = 3, *f* = 2 GHz. Under a Rician fading model, the Rician factor $K_{SU}$ was 1, while $K_{SR}=K_{RU}=\infty$. The reflect link free space path loss factor in Equation (8) was simulated, and the simulation result is shown in Figure 5.

Figure 5 shows the search scheme with the below LoSD in Section 3 to traverse the deployment position of the RIS. It can be seen that the free space path loss factor $\beta_{\mathrm{INDIR}}^{-1}$ of the reflect link is inversely proportional to the path distance product *L*, and the local maximum value is obtained when the horizontal coordinates of RIS are 30 m and 110 m, respectively. From Equations (6) and (7), it is not difficult to verify that the channel gain of the reflect link is at maximum when $\beta_{\mathrm{INDIR}}^{-1}$ is the largest.

**Figure 6:**
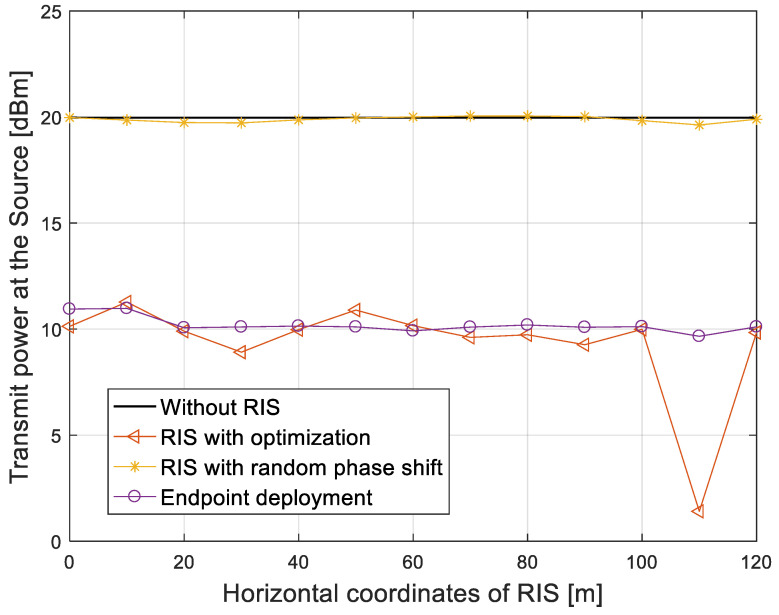
Transmit power at the source versus the horizontal coordinates of RIS.

We considered the transmit power for different RIS positions under the condition of fixed SNR γ = 20 dB. The following schemes were considered: (1) a traditional direct link without RIS; (2) an RIS-aided communication link with an optimal phase shift and LoSD deployment strategy; (3) an RIS-aided communication link with a random phase shift and LoSD deployment strategy; and (4) an RIS-aided communication link with an optimal phase shift and endpoint deployment strategy. The simulation result is shown in Figure 6.

In Figure 6, we compared the transmit power that the source required by all schemes versus the horizontal coordinates of RIS. When the SNR at the user is fixed at 20 dB, the transmit power of the direct link without an RIS is 20 dBm, which is the largest. The transmit power required in the RIS-aided communication link with a random phase shift is about 20 dBm, which is similar to a traditional direct link without an RIS. With the endpoint deployment strategy, the transmit power is reduced to 10 dBm at the horizontal coordinate of 20 m. With the LoSD deployment strategy proposed in this paper, the transmit power has a local minimum when the horizontal coordinates of the RIS are 30 m and 110 m, respectively, and the minimum transmit power can be reduced to 1.4 dBm at 110 m, which is 18.6 dB lower than that of the traditional link without an RIS.

**Figure 7:**
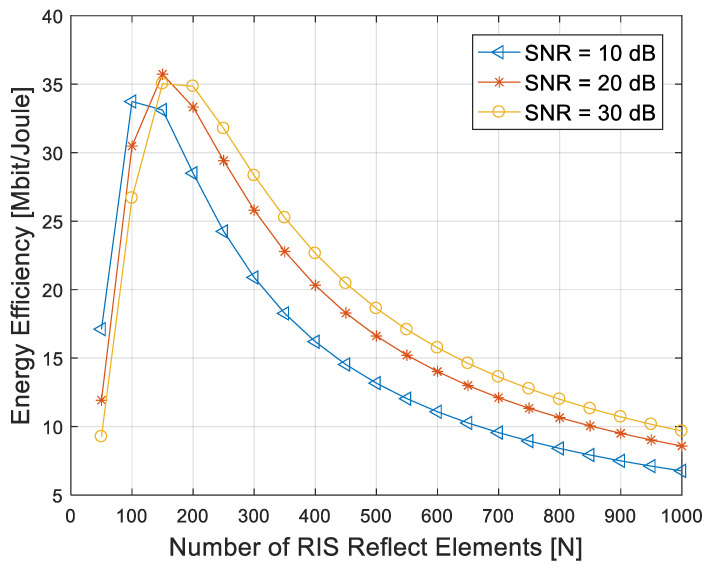
System energy efficiency versus the number of RIS reflect elements with different SNR.

According to Equation (25), the total power consumption of the RIS-aided communication link depends not only on the hardware power consumption of the transceiver, but also on the number of reflect elements. It can be seen that the total power consumption of the system increases with the increase in the number of reflect elements. From Equation (27), we considered the energy efficiency of the system under the condition of a fixed operation bandwidth and SNR. We set *P_s_* = 100 mW, *P_u_* = 10 mW and *P_e_* = 5 mW. The operation bandwidth *B* was 10 MHz, while transmit efficiency η was 0.5. The SNR of the user was fixed at 10 dB, 20 dB and 30 dB, respectively, and the simulation result is shown in Figure 7.

It can be seen in Figure 7 that the energy efficiency of the system first increases and then decreases versus the increase in the reflect elements. In the initial stage, with the increase in the number of reflect elements, the channel gain provided by RIS increases with the increase in *N*. Although the total power consumption of the system increases, the overall energy efficiency of the system still increases. The channel gain that the RIS provided becomes its limit with the continuous increase in the reflect elements, and the power consumption brought by the elements is greater than the channel gain; thus, the system energy efficiency gradually decreases.

With the increase in the fixed SNR at the user, the number of reflect elements needed to maximize the system energy efficiency gradually increases. When the number of reflect elements is the same, the greater the SNR, and the greater the energy efficiency of the system.

## 6. Conclusions

In this paper, we proposed a novel strategy to search for the optimal location of an RIS based on an LoSD. Taking the path loss of the cascade link as the index in a MISO communication link, the optimal location of a single RIS was obtained by traversing the intersection of LoSDs at the source and user ends. Based on an optimal deployment strategy of RIS, the transmit beamforming and phase shift of RIS reflect elements were alternately optimized and compared with different benchmark schemes. The simulation results show that compared with the traditional communication link without RIS and an RIS-aided communication link with random phase shift, the optimized RIS-aided link can reduce the transmission power by at most 18.5 dB. Compared with the endpoint deployment in the ideal scenario, the optimization scheme can reduce the transmit power by at most 8.5 dB.

At the same time, we also discussed the system power consumption and energy efficiency of the RIS-aided communication link in this paper. Different from the communication link without RIS, the total power consumption of the RIS-aided link overlaps the control power consumption of the reflect elements. The system energy efficiency was simulated under the constraints of different SNR. It can be concluded that the system energy efficiency first increases and then decreases with the increase in the number of reflect elements, and the number of reflect elements required to reach the maximum energy efficiency increases with the increase in SNR. The above conclusions have certain reference significance for the deployment of RIS in practical application scenarios and for the maximization of system energy efficiency.

## Data Availability

Not applicable.
